# Enhanced Thermal Stability and Hydrolytic Ability of *Bacillus subtilis* Aminopeptidase by Removing the Thermal Sensitive Domain in the Non-Catalytic Region

**DOI:** 10.1371/journal.pone.0092357

**Published:** 2014-03-14

**Authors:** Xinxing Gao, Zhongmei Liu, Wenjing Cui, Li Zhou, Yaping Tian, Zhemin Zhou

**Affiliations:** Key Laboratory of Industrial Biotechnology, Ministry of Education, School of Biotechnology, Jiangnan University, Wuxi, Jiangsu, China; George Washington University, United States of America

## Abstract

Besides the catalytic ability, many enzymes contain conserved domains to perform some other physiological functions. However, sometimes these conserved domains were unnecessary or even detrimental to the catalytic process for industrial application of the enzymes. In this study, based on homology modeling and molecular dynamics simulations, we found that *Bacillus subtilis* aminopeptidase contained a thermal sensitive domain (protease-associated domain) in the non-catalytic region, and predicted that deletion of this flexible domain can enhance the structure stability. This prediction was then verified by the deletion of protease-associated domain from the wild-type enzyme. The thermal stability analysis showed that deletion of this domain improved the *T_50_* (the temperature required to reduce initial activity by 50% in 30 min) of the enzyme from 71°C to 77°C. The melting temperature (*T_m_*) of the enzyme also increased, which was measured by thermal denaturation experiments using circular dichroism spectroscopy. Further studies indicated that this deletion did not affect the activity and specificity of the enzyme toward aminoacyl-*p*-nitroanilines, but improved its hydrolytic ability toward a 12-aa-long peptide (LKRLKRFLKRLK) and soybean protein. These findings suggested the possibility of a simple technique for enzyme modification and the artificial enzyme obtained here was more suitable for the protein hydrolysis in food industry than the wild-type enzyme.

## Introduction

Aminopeptidases (APs; EC 3.4.11) exist widely in prokaryotic and eukaryotic microbial species, which can selectively catalyze the cleavage of the N-terminal amino acid residues from peptides and proteins. APs are associated with many human diseases and play an important role in a wide range of biological processes [1]–[4]. The research to elucidate the catalytic mechanisms of APs is significant for medicine and pharmacology. APs have great application in various fields because of their broad substrate specificity, strict enantioselectivity, and high thermal stability. Besides being applied to synthesize pharmaceutical compounds and acting as versatile chiral building blocks, they are mainly applied in the food industry [5]. For instance, APs play an important role in debittering protein hydrolysate and increasing the content of free amino acids in protein hydrolysate. They are also used as additives in condiments, such as soy sauce and seasoning blends, to enhance the flavor and the nutritional value of foods [6]–[8].

Recently, we have identified a novel aminopeptidase from *Bacillus subtilis* Zj016 (BSAP) which was most active toward *p*-nitroaniline derivatives of Leu, Arg and Lys, and BSAP exhibited significant sequence similarity to AP encoded by the *ywaD* gene from *B.subtilis* strain 168 but with four different amino acid residues [9]. Moreover, we succeeded in changing its substrate specificity via saturated mutagenesis on the residues located in the substrate binding region [10]. BSAP (MEROPS ID: M28.UPA) belongs to the M28 family which includes bacterial APs and human proteins, such as the prostate-specific membrane antigen and glutamate carboxypeptidase II [3], [11]. In M28 family, *Aeromonas proteolytica* aminopeptidase (AAP), *Streptomyces griseus* aminopeptidase (SGAP) and *Streptomyces septatus* TH-2 aminopeptidase (SSAP) have been extensively studied as the model for understanding the structure and mechanism of other metallopeptidases, which contain two zinc ions in their active sites and share a similar catalytic mechanism. Based on the results from the crystallographic structure and site-directed mutagenesis, the roles of two zinc ions and residues that compose the active center have been elucidated in detail [12]–[17].

Based on the homology modeling and structure analysis of BSAP, we found that there was a protease-associated (PA) domain in the non-catalytic region. The PA domain is about 150 amino acids long, containing two α-helices and seven β-sheets. Because this domain is found in several distinct classes of proteases, it was named PA domain. Besides the different protease families, this domain was also found in two classes of plant transmembrane proteins [18], [19]. However, the role of PA domain remains somewhat elusive. It has been speculated that PA domain was involved in protein-protein interaction [20], [21].

In M28 family, the PA domain was found in only a few APs and the role of PA domain in APs has not been reported yet. Herein we found that the PA domain showed much more flexibility than any other regions in BSAP by using molecular dynamics simulation (MDS). The structure of the hypothetical deleted form of BSAP-ΔPA was modeled and analyzed using MDS, and it was predicted that deletion of this flexible domain can enhance the structure stability. This prediction was supported by the experimental data on thermal stability of BSAP and BSAP-ΔPA. In addition, deletion of the PA domain did not change the activity and specificity toward aminoacyl-*p*-nitroanilines (*p*NAs), but BSAP-ΔPA showed higher catalytic efficiency toward a polypeptide and better hydrolytic ability toward soybean protein than BSAP, indicating the application potential of BSAP-ΔPA in food industry.

## Materials and Methods

### Materials, strains, and plasmids

All aminoacyl-*p*NAs were purchased from Bachem AG. Peptide A (LKRLKRFLKRLK) was purchased from ChinaPeptides Co., Ltd. Plasmid PMA5-BSAP (with the BSAP gene inserted into the *Nde*I-*BamH*I gap of PMA5 [9]) was used for expression of wild-type BSAP and as a template to construct the plasmid for expression of BSAP-ΔPA. *Escherichia coli* JM109 was the host for cloning work. *B. subtilis* WB600 [22] was used as the host strain for gene expression.

### Homology modeling and MDS

Protein domain analysis was performed with the Simple Modular Architecture Research Tool (SMART) on the SMART server (http://smart.embl-heidelberg.de). The tertiary structure of BSAP and BSAP-ΔPA was obtained by homology modeling following the method in our previous study [10], which was used in this study.

MDS was performed using Namd software with charmM force field (http://www.ks.uiuc.edu/Research/namd/). The initial structure was solvated in a cubic box consisting of TIP3P water molecules with a volume of 90×90×90 Å^3^. Na^+^ was also added to neutralize the system. Structure minimization was carried out to remove any unexpected coordinate collision and get the local minima. The whole system was minimized using the descent method plus the conjugate gradient method, and the 150-ps MDS was performed for equilibration at the desired temperature (300 K, 320 K, and 340 K). Finally, a 5-ns MDS was performed on the whole system at the same temperature. An Ewald summation method was used for calculating the total electrostatic energy in a periodic box named Particle Mesh Ewald (PME). The time step of the simulation was set at 2 fs, and the coordinates were saved for analysis every 1 ps.

### Construction of the deleted form BSAP-ΔPA

The deletion of PA domain was performed by inverse PCR using one pair of oligonucleotide primers that was composed of homologous sequences before and after the nucleotides encoding the PA domain (Sense primer: 5- CAACGATTCAACATTCCTGACCGGCTTGAGGGAACACTGTCTTCAGCAGGAACAAACCAAACCTCCCAGAATATCATCGGAATCAAAAA-3; Anti-sense primer: 5- GATGTTCTTTGGT

TTTTTGATTCCGATGATATTCTGGGAGGTTTGGTTTGTTCCTGCTGAAGACAGTGTTCCCTCAAGCCGGTCAGGAA-3.). The PCR program was as follows: 18 cycles of 20 s at 98°C and 9 min at 68°C. The PCR product was treated with *Dpn*I at 37°C for 1 h. Then the PCR product was transformed into competent cells of *E. coli* JM109. After the sequence verified, the extracted plasmid (PMA5-BSAP-ΔPA) was transformed into *B.subtilis* WB600 for expression.

### Expression and purification of BSAP-ΔPA

The recombinant *B. subtilis* harboring plasmid PMA5-BSAP-ΔPA was cultivated in TB medium supplemented with kanamycin (50 μg ml^−1^). After the centrifugation (8000×g, 10 min, 4°C), crude enzyme was obtained from the culture supernatant by salt fractionation with ammonium sulfate (50%–70%, 4°C) and dissolved with buffer A (50 mmol l^−1^ Tris-HCl buffer, pH 8.5). The resultant sample was dialyzed overnight against the same buffer at 4°C and then chromatographed on a Hiprep™ DEAE 16/10 Fast Flow column (GE Healthcare, USA) which was pre-equilibrated with buffer A. A linear NaCl gradient (0-0.6 mol l^−1^) in buffer A was used to elute the enzyme. The active fractions were then subject to Superdex™ 75 10–300 GL column (GE Healthcare, USA). All these procedures were kept under 10°C. The purified enzyme was then used for the subsequent characterization of BSAP-ΔPA. The purified BSAP was prepared following the method of our previous study [9].

### Activity assay and determination of kinetic parameters

Peptide A was dissolved in buffer A, which was prepared at five concentrations as follows: 2, 3, 4, 5, and 6 mmol. 1 μg of BSAP and BSAP-ΔPA was added into 100 μl preincubated substrate solution, respectively. The reaction mixture was incubated at 37°C. Every 5 min after reaction initiation, 10 μl of the mixture was added into 90 μl of 5% acetic acid to stop the reaction. The hydrolysis product was analyzed by an 1100 series Agilent HPLC (Agilent Technologies, USA) equipped with a diode-array detector and Eclipse XDB-C18 column (5 μm, 250×4.6 mm). Gradient elution program was applied for hydrolysis product analysis. The mobile phase was: A (0.1% trifluoroacetic in water) and B (0.1% trifluoroacetic in acetonitrile). The applied elution profile was: 0–16 min, linear gradient from 10% to 80% B; 16–20 min, 100% B isocratic. The flow rate was 1 ml min^−1^ and the temperature was set at 40°C. Peak identification was grounded on retention time data and elution order as compared with the standard under the same conditions. Kinetic parameters were calculated from the reduced amount of the standard.

The molecular weight of each peak was determined by MS analysis. The HPLC-PAD-ESI/MS system was Waters Acquity UPLC chromatography equipped with a Waters Acquity PDA detector and Waters MALDI SYNAPT Q-TOF MS (Waters Corporation, USA). The activity and kinetic parameters toward Leu-*p*NA was determined as our previous study [10].

### Thermal stability assay and circular dichroism (CD) spectrometry

To detect thermal inactivation, the purified enzymes (2 μg ml^−1^) were incubated in the range of 30–80°C. After 30 min, the enzyme was immediately placed on ice for 10 min to decrease the temperature of the sample, and then the remaining activity toward Leu-*p*NA was measured. The remaining activity was recorded as percentage of the original activity.

CD spectroscopy was performed on MOS-450/AF-CD-STP-A (Bio-Logic, France) equipped with a TCU-250 Peltier-type temperature-control system. The unfolding curves were measured at 222 nm, from 40 to 86°C, using the temperature scan mode with a gradient of 1°C min^−1^. The measurements were performed in buffer A, with a protein concentration of 0.1 mg ml^−1^, using a 1 mm path-length quartz cuvette.

### Hydrolysis of soybean protein

Soybean protein was prepared with deionized water at the final concentration of 5% (w/v). After heated at 90°C for 10 min, the pH of the solution was adjusted to 8.5 by NaOH. In order to improve hydrolysis efficiency, alkaline protease (1 g l^−1^) was added and treated at 50°C for 5 h. Then the purified BSAP and BSAP-ΔPA (0.1 g l^−1^, finial) were used to hydrolyze soybean protein at 50°C for 5 h, respectively. The reaction without AP was used as the control.

The degree of hydrolysis (DH) was defined as the percentage of free amino groups cleaved from protein, which was calculated from the ratio of α-amino nitrogen to total nitrogen. The α-amino nitrogen content was determined by formaldehyde titration method [23]. The total nitrogen content was determined by Kjeldahl method [24]. The peptide molecular mass distribution was determined by high-performance gel filtration chromatography with the TSKgel G2000 SWXL column (300×7.8 mm, Tosoh, Japan).

## Results

### Sequence and structure analysis of BSAP

**Figure 1:**
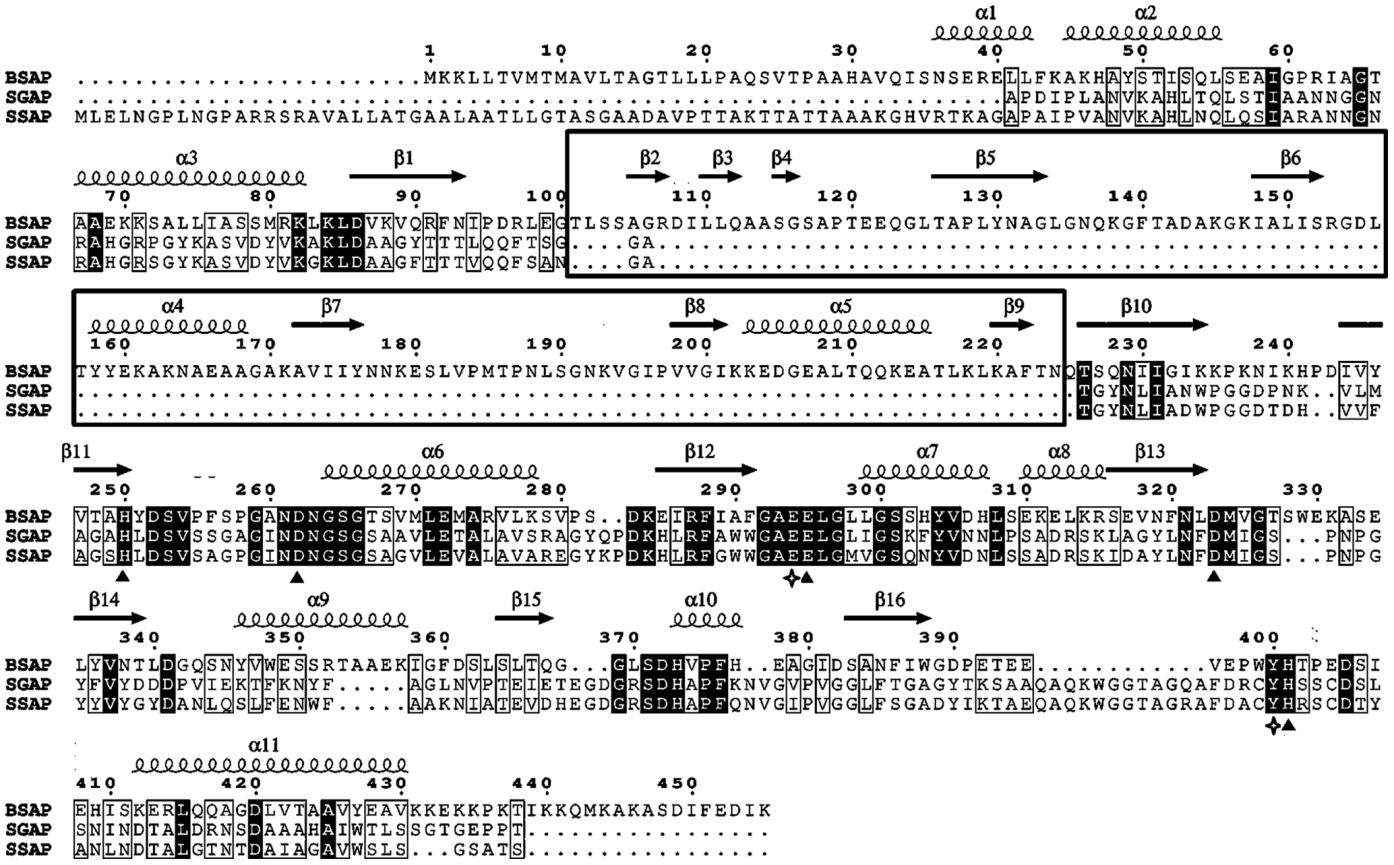
Sequences alignment of BSAP, SGAP, and SSAP. BSAP, *Bacillus substilis* aminopeptidase; SGAP, *Streptomyces griseus* aminopeptidase; SSAP, *Streptomyces septatus* TH-2 aminopeptidase. All sequences are full-length. The α helices and β sheets of BSAP were indicated by α 1–11 and β 1–16. The box indicates the PA domain (α 4–5 and β 2–9). The filled triangles indicate the five residues in metal-binding sites, and the open tetragons indicate the two catalytic residues. The alignment was performed with COBALT and the figure was produced with ESPript.

**Figure 2:**
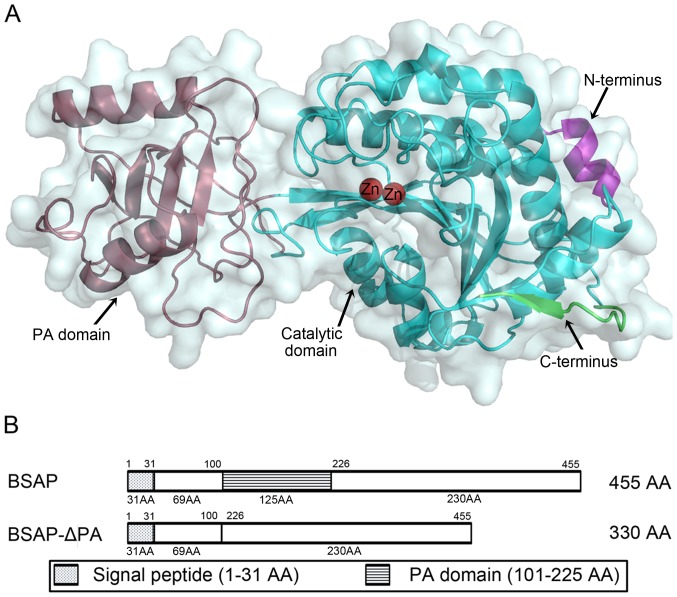
Structure analysis of BSAP. A: The predicted structure of BSAP. The structure was obtained by homology modeling, using AP from *Aneurinibacillus* sp. strain AM-1 (PDB ID. 2EK8) as the template. The PA domain (raspberry), catalytic domain (cyan), zinc atoms (red), N-terminus (purple), and C-terminus (green) were labelled, respectively. B: Schematic domain structures of full-length BSAP and BSAP-ΔPA.

The complete BSAP contains 455 amino acids and the calculated molecular mass is 49.4 KDa (Fig. 1). SMART analysis showed that BSAP contained a signal peptide (1 –31 aa) as well as a PA domain (101 –225 aa). However, the PA domain was not found in the well-studied APs in M28 family, such as SGAP, AAP, and SSAP. The amino acids that are part of the catalytic domain in these APs are highly conserved and are found in BSAP (Fig. 1). The tertiary structure of BSAP (Fig. 2A) showed that PA domain existed in the non-catalytic region and was positioned as a “lid” covering the catalytic region of BSAP.

**Figure 3:**
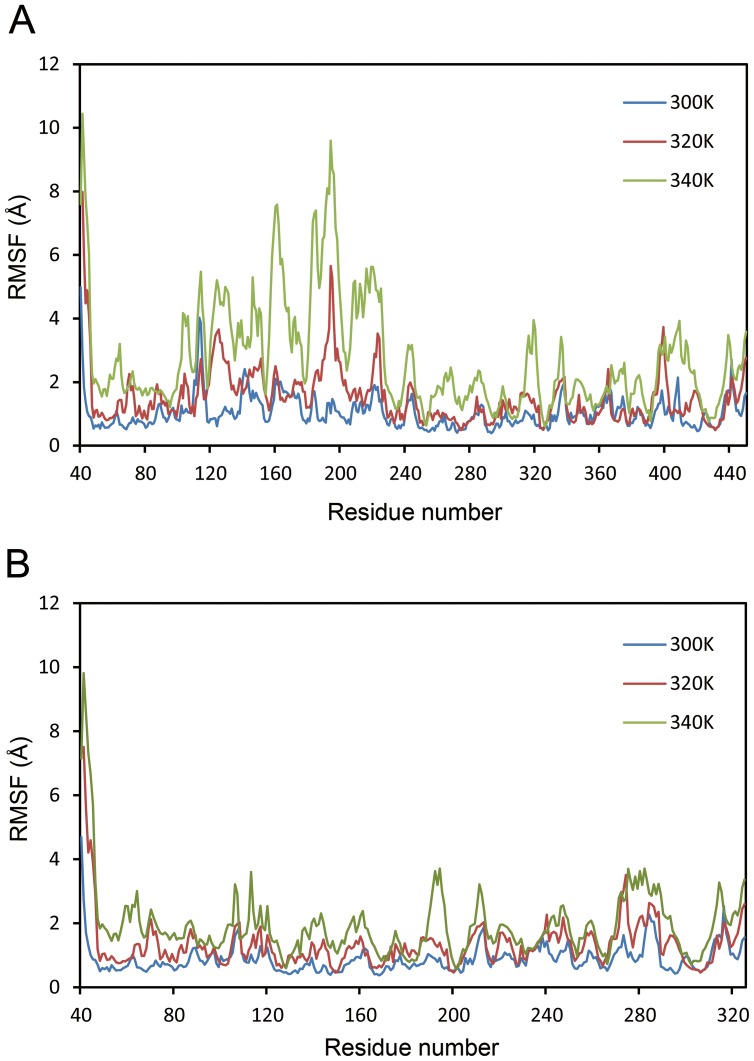
RMSF values of BSAP and BSAP-ΔPA at various temperatures. A: RMSF values of BSAP. B: RMSF values of BSAP-ΔPA. The residues in the structure of BSAP-ΔPA were renumbered from 1 to 330 after deleting the PA domain. The different temperatures were indicated by blue (300 K), red (320 K), and olive (340 K), respectively.

MDS was carried out to identify the conformational flexibility of PA domain of BSAP by calculating the root mean square fluctuation (RMSF) values of backbone atoms. Average RMSF values reflect fluctuation at individual residues, and a higher RMSF value indicates less stability [25]–[27]. By MDS at multiple temperatures, RMSF values of backbone atoms against each residue were calculated over the last 1000 ps for both BSAP and BSAP-ΔPA. As shown in Fig. 3A, RMSF values of the residues of PA domain (101–225) increased significantly with the increase of temperature, which suggested that PA domain was a thermal sensitive region. In the case of BSAP-ΔPA, the MDS data indicated that the degree of variability of the structure was relatively low as the treatment temperature rose, suggesting that deletion of this flexible domain resulted in a stable conformation (Fig. 3B). Therefore, we speculated that removing this flexible domain was advantageous to enhance the thermal stability.

### Thermal stability of BSAP and BSAP-ΔPA

To verify this speculation, the PA domain was deleted (Fig. 2B) and its deleted form (BSAP-ΔPA) was over-expressed in *B.subtilis* and purified. The purified BSAP was used as the control.

**Figure 4:**
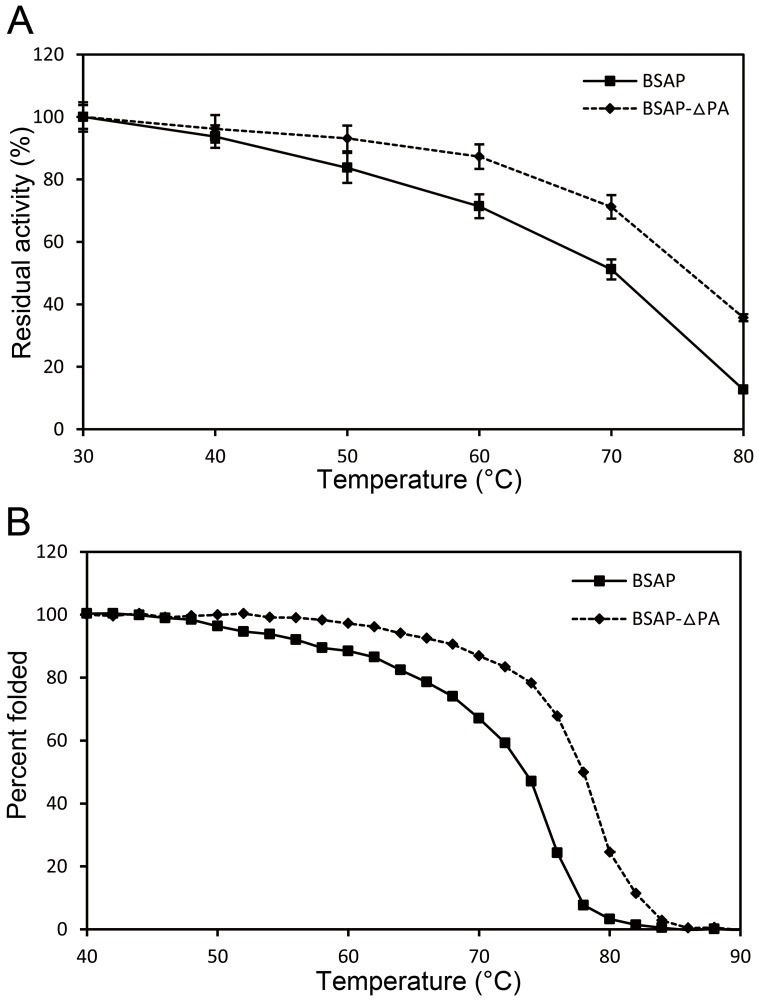
Effect of temperature on the stability of BSAP and BSAP-ΔPA. A: Thermal stability of the enzymes was determined by monitoring residual enzymatic activity after incubating for 30 min at various temperatures. Data points correspond to the mean values of three independent experiments. B: Temperature-induced unfolding measured using CD spectroscopy of BSAP and BSAP-ΔPA.

The thermal stability of BSAP and BSAP-ΔPA was determined by measuring residual activity after incubation for 30 min at various temperatures (Fig. 4A). The temperature at which BSAP lost 50% of its activity (*T_50_*) was approximately 71°C. The deletion of PA domain increased the apparent *T_50_* to 77°C, 6°C higher than that of BSAP. To further investigate the thermal stability of these two enzymes, we performed thermal denaturation experiments using CD spectroscopy. The thermal stability of them was quantified by deriving an apparent melting temperature (*T_m_*) from the CD-unfolding curves. As shown in Fig. 4B, this value for BSAP-ΔPA was determined to be 78°C, which was higher than that of BSAP (74°C). These findings indicated that the deletion of PA domain improved the thermal stability of BSAP.

### Effect of the PA domain deletion on catalytic efficiency of BSAP

**Figure 5:**
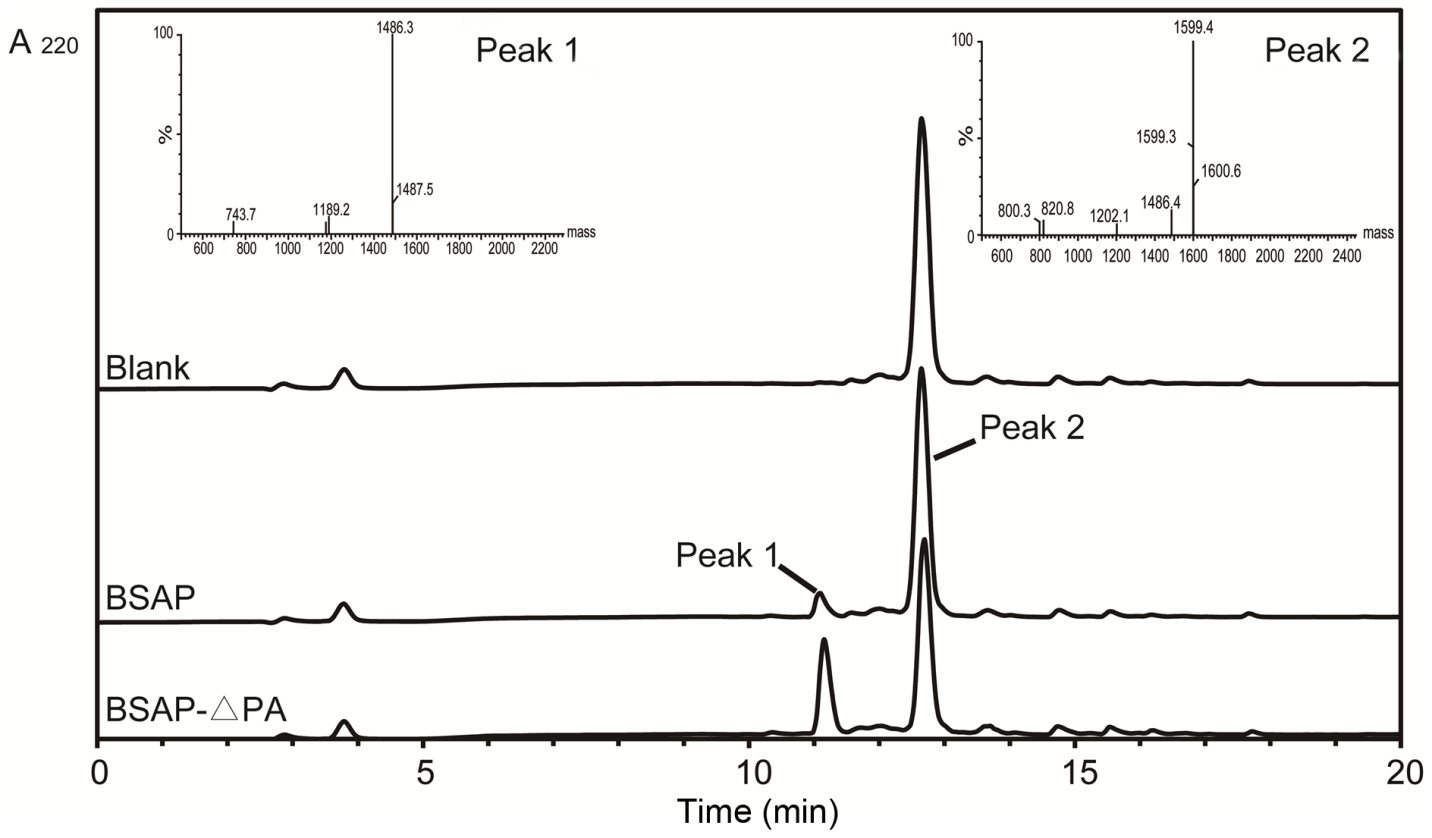
HPLC chromatogram and mass spectrum of the hydrolysate from Peptide A. Peptide A (4 mmol) were hydrolyzed by BSAP and BSAP-ΔPA at 37°C for 30 min. The reaction without the enzyme was performed as the control. The insets show the mass spectrum of Peak 1 and Peak 2.

Compared with BSAP, BSAP-ΔPA showed similar hydrolytic activity toward Leu-*p*NA (Table 1) and same specificity toward aminoacyl-*p*NAs (most active toward Leu-*p*NA, followed by Arg-*p*NA, Lys-*p*NA, and Met-*p*NA), suggesting that the PA domain did not participate in the catalytic process. Based on this, we synthesized Peptide A (LKRLKRFLKRLK) to investigate the effect of PA domain on hydrolytic activity toward macromolecular substrate. Fig. 5 represents the HPLC chromatogram and mass spectrum (MS) of the hydrolysate from Peptide A. As shown, the hyrolysate was eluted as two major peaks, one peak with a small molecular mass (Peak 1, 1486 Da) and another peak corresponded to the Peptide A (Peak 2, 1599 Da). The peak 1 is estimated to be the calculated mass of Peptide B (KRLKRFLKRLK), suggesting the first residue (Leu) of Peptide A was efficiently cleaved. The results indicated that BSAP and BSAP-ΔPA cleaved the first residue (Leu) from N-terminus of Peptide A at different rates.

**Table 1:** Kinetic parameters of BSAP and BSAP-ΔPA a.

Substrate	BSAP (-ΔPA)	*k*_cat_ (s^−1^)	*K*_m_ (mmol)	*k*_cat_/ *K*_m_ (mmol^−1^ s^−1^)
Leu-*p*NA	BSAP	70.66±3.58	2.52±0.11	28.1
	BSAP-ΔPA	62.68±2.89	2.43±0.23	25.8
Peptide A	BSAP	2.79±0.16	7.78±0.37	0.359
	BSAP-ΔPA	7.41±0.42	3.32±0.18	2.232

a *K*_m_ and *k*_cat_ values were calculated by non-linear regression analysis. The values are means ± standard deviations of three independent experiments.

We also characterized the kinetics of BSAP and BSAP-ΔPA toward Leu-*p*NA and Peptide A, and the results were summarized in Table 1. In the case of Leu-*p*NA, BSAP and BSAP-ΔPA showed the similar hydrolytic efficiency, and there were no significant differences between the kinetic parameters of them. In contrast, for Peptide A hydrolysis, the *k*_cat_ and *K*_m_ values of BSAP-ΔPA were 2.7-fold higher and 2.4-fold lower than those of BSAP, respectively. These indicate that deletion of the PA domain does not affect the catalytic efficiency toward aminoacyl-*p*NAs, but the catalytic efficiency toward the Peptide A is greatly improved by this deletion.

### Comparison of the hydrolytic ability of BSAP and BSAP-ΔPA toward soybean protein

**Figure 6:**
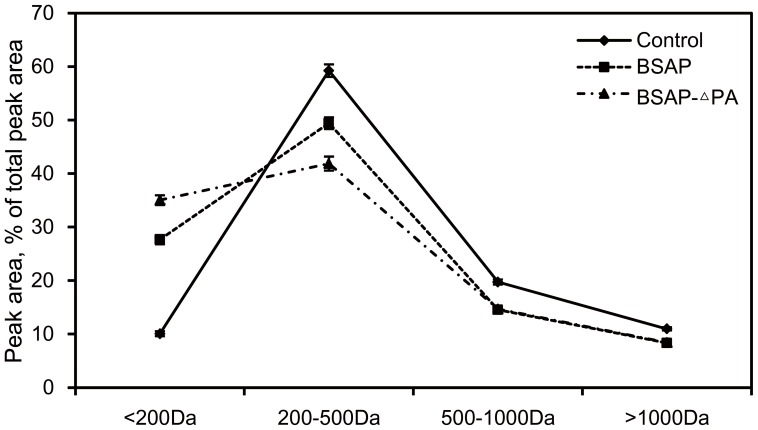
The peptide molecular mass distribution estimates for the hydrolysate of soybean protein. The reaction without AP was used as the control. The values present the means of three independent experiments.

To compare the utility of these two enzymes, soybean protein was selected as the substrate. The peptide molecular mass distribution and degree of hydrolysis (DH) were determined, respectively. As shown in Fig. 6, the addition of BSAPs (BSAP and BSAP-ΔPA) increased the concentration of free amino acids (< 200 Da) significantly by reducing the concentration of peptide (200–1000 Da). This concentration differential was consistent with the DH of each group, 9.6% of the control, 33.2% of BSAP, and 44.7% of BSAP-ΔPA. Compared with that of BSAP group, although the ratio of peptide (> 500 Da) in BSAP-ΔPA group changed negligibly, an apparent reduction in the ratio of peptide (200–500 Da) and the consequent increase in the ratio of peptide (< 200 Da) were observed. These results suggested that BSAP-ΔPA was more conductive for protein hydrolysis than BSAP.

## Discussion

In vivo, the enzymatic reaction is an important biological process that can serve a wide variety of purposes [28]. Inappropriate activity of enzymes can be deleterious to the cell or the organism that produces them [29]. Thus, many enzymes contain conserved domains that can serve as an auto-inhibitor, a substrate-targeting domain or a regulatory domain, allowing catalysis events to occur only in the correct subcellular compartment, at the correct time and with the correct substrates [30]. However, in the industrial application of the enzymes, sometimes these regulatory domains were unnecessary or even detrimental to the catalytic process. In order to satisfy the industrial requirement, it was an efficient approach to reconstruct the enzymes via deletion of these redundant regions. It has been reported that truncation of the cellulose binding domain of glucanase improved its thermal stability [31]. Recently, Xiangtao Liu et al [32] reported that N-terminal truncation of a maleate *cis*-*trans* isomerase resulted in a highly active enzyme for the biocatalytic production of fumaric acid. The research about changing optimum pH of an invertase by N-terminal truncation was also reported, which made it suitable for industrial application [33]. In this study, based on homology modeling and MDS, we found that the PA domain of BSAP was a thermal sensitive region, and predicted that removing this flexible domain was able to enhance the thermal stability. This prediction was then supported by the experimental data.

The flexible PA domain was positioned as a “lid” covering the catalytic region of BSAP (Fig. 2A). Highly flexible regions represent an important part of the protein structure and play an important role in protein function, stability, and folding [34]. In the past decades, there are a lot of researches about improving the thermal stability of enzymes by reducing the conformational flexibility in the non-catalytic region [35]–[37]. It was generally recognized that the reduced flexibility may decrease the entropy during protein unfolding by reducing the numbers of unfolded conformations [38]. Deletion of this flexible domain from BSAP can reduce the structure flexibility to some degree, resulting in the enhanced thermal stability.

In addition to the stability, the active site was exposed by removing this “lid”, which made it more possible to interact with the N-terminus of macromolecular substrate. This could explain why BSAP-ΔPA exhibited higher catalytic efficiency toward the Peptide A than BSAP. To be noted, the first residue (Leu) of Peptide A was efficiently cleaved to form Peptide B, but the further hydrolysis of Peptide B was not detected during this process. It may be related to the following reasons: first, the concentration of Peptide A was much higher than that of Peptide B in this process, and the substrate competition of Peptide A strongly inhibited the hydrolytic activity of Peptide B; second, since the hydrolytic activity of BSAP-ΔPA toward Leu–*p*NA was much higher than Arg-*p*NA, BSAP-ΔPA might be prone to hydrolyze the peptide with Leu residue at the N-terminus. Kinetic analysis revealed that deletion of the PA domain affected both *K*_m_ and *k*_cat_ values (Table 1). Thereby we speculated that the existence of PA domain not only interfere with the substrate access but also lead to a subtle orientation shift of the bound substrate. This shift may change the distance between the substrate and the catalytic residues, which resulted in a higher *k*_cat_ value toward Peptide A.

In the experiment of soybean protein hydrolysis, BSAP-ΔPA exhibited better hydrolytic ability than BSAP, and the difference of the peptide molecular mass distribution between these two groups was observed in the peptide smaller than 500 Da but not the peptide larger than 500 Da. This result did not exhibit the higher hydrolytic efficiency of BSAP-ΔPA toward the macromolecular substrate than that of BSAP. In this case, we found that the peptide (200–500 Da) was approximately 60% of total peptides after the hydrolysis by alkaline protease, and as shown in Table 1, the hydrolytic efficiency toward the analogue of the dipeptide was much higher than that toward Peptide A. Due to these findings, the enzyme was liable to hydrolyze the small peptide during the hydrolytic process.

In conclusion, we succeeded in enhancing the thermal stability of BSAP by removing the thermal sensible domain. Further studies showed that BSAP-ΔPA possessed better hydrolytic ability toward soybean protein than the wild-type enzyme, exhibiting high application potential for the protein hydrolysis in food industry. In this study, we proved that the PA domain did not participate in the catalytic process of BSAP. Because the function of PA domain in APs is still unknown, these findings will be useful for the further research on the physiological function of PA domain.
